# Dietary phenethylisothiocyanate attenuates bowel inflammation in mice

**DOI:** 10.1186/1472-6769-10-4

**Published:** 2010-04-27

**Authors:** Moul Dey, Peter Kuhn, David Ribnicky, VummidiGiridhar Premkumar, Kenneth Reuhl, Ilya Raskin

**Affiliations:** 1 Department of Nutrition, Food Science and Hospitality, South Dakota State University, Box 2275A, Brookings, SD 57007, USA; 2 Phytomedics Inc., 1085 Cranbury South River Road, Jamesburg, NJ 08831, USA; 3 Rutgers University, Biotechnology Center, 59 Dudley Road, New Brunswick NJ 08901, USA; 4 Rutgers University, Department of Pharmacology & Toxicology, Ernest Mario School of Pharmacy, Piscataway, NJ 08854, USA; 5 Department of Cancer and Cell Biology, University of Cincinnati, Cincinnati, OH 45219, USA

## Abstract

**Background:**

Phenethylisothiocyanate (PEITC) is produced by Brassica food plants. PEO is a **P**EITC **E**ssential **O**il containing >95% natural PEITC. PEITC is known to produce various health benefits but its effect in alleviation of ulcerative colitis signs is unknown.

**Results:**

In two efficacy studies (acute and chronic) oral administration of PEO was effective at remitting acute and chronic signs of ulcerative colitis (UC) in mice. Disease activity, histology and biochemical characteristics were measured in the treated animals and were compared with appropriate controls. PEO treatment significantly improved body weights and stool consistency as well as decreased intestinal bleeding. PEO treatment also reduced mucosal inflammation, depletion of goblet cells and infiltration of inflammatory cells. Attenuation of proinflammatory interleukin1β production was observed in the colons of PEO-treated animals. Expression analyses were also carried out for immune function related genes, transcription factors and cytokines in lipopolysaccharide-activated mouse macrophage cells. PEO likely affects an intricate network of immune signaling genes including a novel concentration dependent reduction of total cellular Signal Transducer and Activator of Transcription 1 (STAT1) as well as nuclear phosphorylated-STAT1 (activated form of STAT1). A PEO-concentration dependent decrease of mRNA of C-X-C motif ligand 10 (a STAT1 responsive chemokine) and Interleukin 6 were also observed.

**Conclusions:**

PEO might be a promising candidate to develop as a treatment for ulcerative colitis patients. The disease attenuation by PEO is likely associated with suppression of activation of STAT1 transcription and inhibition of pro-inflammatory cytokines.

## Background

Inflammatory bowel disease (IBD), affecting an estimated two million people annually in the US [1,2], is predominantly comprised of Crohn's disease (CD) and ulcerative colitis (UC). IBD is a set of chronic and relapsing inflammatory disorders of the intestine caused by multifactorial conditions in a genetically predisposed individual. UC primarily affects the mucosal lining of the colon and rectum, whereas CD may extend to any part of the gastrointestinal tract and is characterized by transmural inflammation [2,3]. Since the etiology of both CD and UC remains unclear, successful treatment strategies targeting wider sections of the population have not been found. In recent years meta-analyses of clinical trials have been performed in several instances to establish efficacy of antibiotic and other therapies. Non-steroidal anti-inflammatory drugs such as Asacol, and immune-suppressive agents are commonly used to treat IBD alone or in combination with short-term antibiotic therapies [4-6]. While adjunct antibiotic therapies may be useful [6], their long term use is not common for reasons beyond the scope of discussion here. Other oral therapies generally for long term remission and to prevent flare-up, to which not all patients adequately respond, are often combined with surgical interventions of the colon. Patients with prolonged UC are at an increased risk for developing colitis-associated cancer (CAC) [7]. Long-term use of some IBD therapies can reduce the risk of CAC by 75-81% in responding patients [8]. Side effects of existing treatments, such as kidney damage from long-term use of mesalamine (Asacol) or increased risk of infections from use of immune suppressive agents are common [4,5]. The lack of efficacious drugs to treat the general patient population, combined with the prevalence of negative side effects, emphasizes the need for the development of new, effective, and well-tolerated anti-inflammatory therapies for UC. Alternative treatments, including botanical therapies, have been proposed [9]. Unfortunately, objective evidence of consistent efficacy and safety of botanical products is generally nonexistent, despite their gain in public acceptance [9].

A combination of genetic susceptibility factors and an altered immune response driven by enteric microbial factors and oxidative stress, contributes to the initiation and chronification of IBD as suggested by various animal models [10,11]. Chemically induced intestinal inflammation is among the most commonly used IBD animal models, as the onset of inflammation is immediate and the procedure is relatively straightforward. Among the chemical models, the dextran sodium sulfate- (DSS) induced model of bowel inflammation can be adapted for the study of both acute and chronic colitis and can be extended for the study of CAC [11]. In addition, the DSS-induced model recreates some important immunological and histopathological aspects of IBD occurring in humans.

Isothiocyanates (ITCs) are naturally occurring sulfur-containing compounds commonly found in Brassicaceae family vegetables such as cabbage, cauliflower, watercress and broccoli [12]. Phenethylisothiocyanate (PEITC) is a well-studied ITC, occurring naturally in the form of its glucosinolate precursor, gluconasturtiin. *Barbarea verna *is a Brassicaceous herb that is used in salads, soups, and garnishes and in our study is one of the richest sources of dietary PEITC, with the highest levels contained in the seeds [13]. PEITC showed chemopreventive activity against various cancers [13,14] and no apparent toxicity even when administered in high doses in rats and dogs as determined by NOEL (no-observed-adverse-effect-level) in drug safety studies [15]. It is also known for other beneficial effects such as anti-oxidative and anti-inflammatory activities [16-19]. PEITC inhibited Nuclear Factor kappaB (NFκB) activity in colon cancer cells and macrophages [16-18,20]. Our previous study [19] showed that PEITC was the most effective of several ITCs tested in suppressing expression of NFκB-regulated proinflammatory genes. In the same study PEO (PEITC essential oil, >95% natural PEITC from *B. verna*) had strong anti-inflammatory activity, reducing paw edema in rats with an effect that was comparable to that of the reference drug aspirin administered at the same concentration [19]. Research shows that PEITC is stable in biological samples and has high oral bioavailability in rats [21]. Here, we report the efficacy of PEO in alleviating clinical signs of DSS-induced acute and chronic colitis in mice. Using in vivo and in vitro models, we have measured effects of PEO on disease activity index, histological parameters, and changes in proinflammatory cytokine levels as well as changes in expression and activation of a key transcription factor, Signal Transducer and Activator of Transcription (STAT1). To the best of our knowledge no prior report has evaluated the anti-inflammatory effects of PEITC on IBD in mammalian models.

## Methods

### Chemicals and Biochemicals

Antibiotics, dimethyl sulphoxide (DMSO), LPS (lipo-polysaccharide from *E.coli*, serotype 055:B5) and PEITC standard were purchased from Sigma Chemicals (St. Louis, MO), 5-amino salicylic acid (5-ASA, also known as mesalamine) from TCI America (Portland, OR) and DSS from MP Biomedicals (Solon, OH). All other chemicals, including cell culture media, were obtained from Invitrogen Inc. (Carlsbad, CA). Reagents used in quantitative PCR, including enzymes, were supplied by Stratagene Inc. (La Jolla, CA), cress seeds (certified non-contaminated) from Kitazawa Seed Company (Oakland, CA) and RAW 264.7 cell line (ATCC TIB-71) from American Type Culture Collection (Manassas, VA). All antibodies were obtained from Santa Cruz Biotechnology Inc (Santa Cruz, CA), unless otherwise mentioned.

### Preparation of PEO

PEO (containing >95% PEITC) was extracted by hydro distillation of ground winter cress seed as was previously described in Ribnicky et al., [13] and Dey et al., [19]. Gas chromatography/mass spectroscopy (GC/MS) was used to confirm the purity of the obtained PEO. The samples were injected into a Hewlett-Packard mass spectrometer (model 5890/5971) equipped with a 30-m × 0.25 mm DB-5 MS fused silica capillary column (J&W Scientific, Folsom CA). Chromatographic parameters were as previously described [13,19]. The retention time of PEITC is 11.3 min. The major ion of PEITC has a mass of 91 (m/z) and molecular ion of mass 163 (m/z). The abundance of these ions and the integration value of the entire peak were used together with standard curves created previously from a PEITC chemical standard (Sigma Chemicals, St. Louis, MO) to quantify the PEITC in PEO.

### In vivo animal studies

All studies were performed in accordance with the Guide for the Care and Use of Laboratory Animals as adopted and promulgated by the U.S. National Institutes of Health. C57BL/6J male mice, seven weeks old, were used. The mice were housed in a room maintained at a constant temperature of 24-26°C with 12-h light/dark cycle and had free access to food (Purina chow) and drinking water. Before the start of experiments, one week was allowed for their acclimatization to these conditions. Two murine models of colitis, the chronic and acute, were used following Aharoni et al., [5] and Wirtz et al., [11]. The mice were randomized into four groups, which are described in Table 1. For the acute and chronic models, each of the groups, except healthy controls, consisted of 6 and 10 animals respectively, housed 3-4 per cage. The healthy control (water group) consisted of 4 animals in both experiments. Development (and variation) of clinical signs was not anticipated in this group, hence the small number was determined to be sufficient. Treating this group with vehicle helped to account for any stress among animals due to daily oral gavage.

**Table 1:** In vivo experimental set-up for acute and chronic colitis models

Groups	Acute model (5 days induction, ad libitum)	Chronic model (36 days induction,, ad libitum)	Treatment (14 days, once daily)	Comment
Water	Tap water	Tap water	Vehicle (9% DMSO+corn oil)	Healthy control
DSS	3% DSS	2.5% DSS (3 days) followed by tap water (6 days), 4 cycles	Vehicle (9% DMSO+corn oil)	Diseased control*
PEO	3% DSS	2.5% DSS (3 days) followed by tap water (6 days), 4 cycles	PEO 75 mg/kg (>95% PEITC)	Unknown test group
5-ASA	3% DSS	2.5% DSS (3 days) followed by tap water (6 days), 4 cycles	5-ASA 50 mg/kg	Reference test group

**Figure 1:**
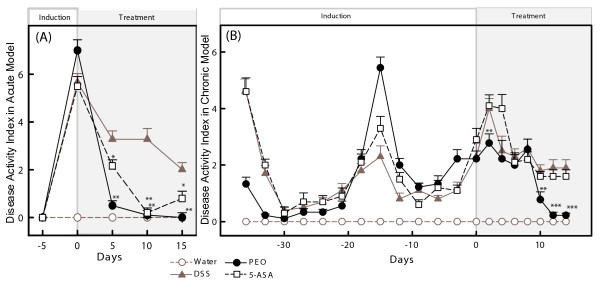
**Effects of orally administered PEO and 5-ASA on the Disease activity index (DAI) in DSS-induced colitis**. Experimental mouse groups and disease induction are defined in Table 1 and scoring criteria in Table 2. Significance of treatments in PEO and 5-ASA groups in respect to DSS group and are indicated by *, *p *< 0.05 and **p < 0.01 and ***p < 0.001. (A) Acute colitis model. Data as mean ± SEM (n = 6) are shown at five-day intervals. (B) Chronic colitis model. Data as mean ± SEM (n = 10) are shown at three-and two-day intervals during induction and treatment periods respectively.

**Figure 2:**
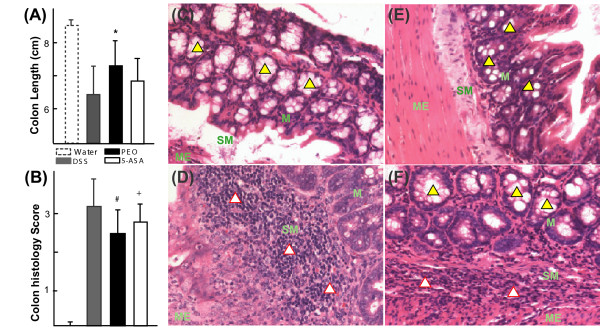
**Effects of orally administered PEO and 5-ASA on colon length and histopathology**. Experimental groups (chronic) and disease induction are defined in Table 1. (A) The DSS receiving groups had shorter average colon length as compared to water group (healthy controls) (n = 10). The shortening was relieved significantly in PEO treated group with respect to DSS group (*, *p *< 0.05). (B) Histogram showing blinded histo-pathological scores (n = 5; #, *p *= 0.07; +, *p *= 0.08) (C) Representative section (×200) from healthy (water) control group showing intact goblet cells and absence of inflammatory cells (D) Representative section (×200) from DSS group showing massive inflammatory cell aggregates and extensive loss of goblet cells (E) Representative section (×200) from PEO-treated group showing partial loss of goblet cells but absence of inflammatory cell accumulation (F) Representative section (×200) from 5-ASA-treated group showing minor loss of goblet cells and moderate infiltration of inflammatory cells. M, mucosa; SM, submucosa; ME, muscularis; Goblet cells are shown with yellow triangles with black borders; Inflammatory cells are marked with white triangles with red border. Histopathology was analyzed from H&E stained colon sections (6 μm).

* Statistical significance of treatments of PEO and 5-ASA groups are shown with respect to DSS group, unless otherwise mentioned

1. Acute model of colitis: Acute ulcerative colitis was induced by freshly prepared 3% DSS (ad libitum) in drinking water for five days (Table 1). In a separate experiment we had previously tested various DSS concentrations (2-4%) for acute disease development (data not shown). At a concentration higher than 3% DSS, some deaths occurred that corresponded with severity of clinical signs. Therefore, we used the highest tolerated concentration of 3% for our actual experiment. Mice were observed individually once a day for their general health conditions, including body weight, diarrhea, rectal bleeding and inflammation and rectal prolapse. Occult bleeding was tested daily in stool per cage using a commercial ColoScreen Kit (Helena Laboratories, Beaumont, TX). Disease activity index (DAI) (Fig 1) was determined following the scheme shown in Table 2. On the 15^th ^day post-treatment, mice were sacrificed using carbon dioxide inhalation. Colons were collected terminally, measured for their gross length, and flushed with sterile phosphate-buffered saline without calcium and magnesium (Gibco Carlsbad, CA) containing 20 μg/ml gentamycin (Sigma Chemicals, St Louis, MO). Entire colons were then stored in histology cassettes immersed in 10% neutral buffered formalin. Paraffin cross-sections (6 μm) were stained with hematoxylin and eosin (H&E) for histological evaluation (×200 magnification) of colonic damage. Histopathological assessment was performed in a blinded manner following the published scoring system [11] (Table 3, Fig 2).

**Table 2:** Disease Activity Index (DAI) to assess clinical colitis in mice (modified based on Aharoni et al., 2006; Wirtz et al., 2007)

Score	Body wt change (%)	Internal intestinal bleeding, FOBT test strip (colorimetric)	Rear end visible bleeding+ inflammation	Stool consistency	*Rectal prolapse
0	Gain, loss <1	No color change	Negative	Normal	None
1	Loss 1-5	-	Either one, mild	-	-
2	Loss 5-10	Color change	Either one, severe	Loose	Observed
3	Loss 10-15	-	Both, mild	-	
4	Loss >15	-	Both, severe	Diarrhea	-

* Rectal prolapse was only observed in the chronic model

**Table 3:** Scoring system for inflammation-associated histological changes in the colon (Wirtz et al., 2007)

Score	Histologic changes
0	No evidence of inflammation
1	Low level of inflammation with scattered infiltrating mononuclear cells (1--2 foci)
2	Moderate inflammation with multiple foci
3	High level of inflammation with increased vascular density and marked wall thickening
4	Maximal severity of inflammation with transmural leukocyte infiltration and loss of goblet cells

2. Chronic model of colitis: Chronic colitis was induced by four cycles of 9 days of alternating DSS and tap water (ad libitum) (detailed in Table 1). Each 9-day cycle consisted of 3 days of 2.5% DSS, followed by 6 days of tap water. Treatment regimens and sacrifice of mice were the same as in acute model except that necropsy was carried out on day 16. Five samples from each group, except water group, were collected for histopathology as described for the acute model, and remaining five were collected for full thickness organ culture as described by Wirtz et al., [11]. Similarly for the control water group, two were collected for full thickness organ culture and two for histopathology. All clinical assessments were performed as described for acute model above.

### Organ culture and ELISA

**Figure 3:**
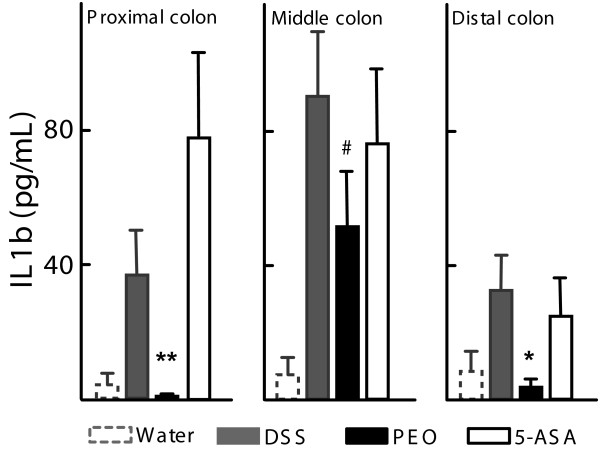
**Effect of orally administered PEO and 5-ASA on proinflammatory Interleukin 1β (IL1β) expressed in mouse colons**. Experimental groups and disease induction are defined in Table 1. Data are means ± SEM (n = 5), *, *p *< 0.05, **, *p *< 0.01, #, *p *= 0.06. Protein expression in the gut was measured using ELISA.

Two biopsy punches were terminally collected from each of the proximal middle and distal parts of individual colons using a 3 mm dermal punch biopsy instrument (Miltex, York, PA). Each biopsy specimen was transferred into separate wells of a 48-well microtiter plate containing 500 μl of supplemented RPMI1640 media (Gibco, Carlsbad, CA) and incubated at 37°C for 12 hours in a cell culture incubator [11]. Supernatants were collected and stored at -20°C until further use. Interleukin1β (IL1β) protein levels released by the biopsy samples in the media were assessed with a quantitative enzyme-linked immunosorbent assay kit (ELISA) (Bender Med Systems, Burlingame, CA), according to manufacturer's instruction, and the values were expressed as pg/ml (Fig 3).

### Macrophage Cell Culture Assay

The mouse monocyte/macrophage cells, RAW 264.7 (ATCC TIB-71), were cultured and treated as previously described by Dey et al., [19].

### Cell Viability Assay and Dose Range Determination

A Cell Titer 96 MTS assay kit (Promega Corp., Madison, WI) was used to determine the relative number of viable cells remaining after incubation with treatments. For actual in vitro experiments with PEO, the highest non-toxic concentration or less was selected.

### Total RNA Extraction, Purification, and cDNA Synthesis

Total RNA extraction, purification and cDNA synthesis were performed as described previously in Dey et al., [19].

### Real Time Quantitative PCR and Gene Array

**Figure 4:**
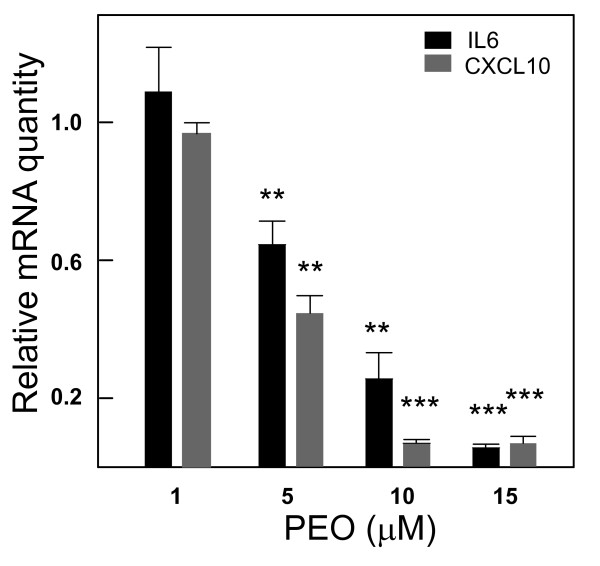
**Concentration dependent mRNA expression of IL6 and CXCL10 (selected novel hits presented in Table 5) in response to PEO treatments in LPS-activated mouse macrophage**. Effect of PEO treatment was measured by the relative mRNA quantity of the genes of interest in the treated cells with respect to that of LPS activation only (positive control) that is normalized to a value of 1.00; β actin served as an internal control. Lower values represent greater inhibitory effects with 0.00 corresponding to a complete inhibition of the induced gene expression; Values are mean ± S.E. **, ***p ***< 0.01; ***p < 0.001. The gene expression was amplified using Real time RT-PCR.

Q-RT-PCR was performed as previously described [19]. Gene-specific primers (IDT Inc., Coralville, IA) used in the current study are described in Table 4. For gene array experiments (Table 5), PCR-arrays (APM_025, SABiosciences Inc, MD) were purchased and manufacturer's protocol was followed. Relative quantification using SYBR green technology and standard ΔΔCt method was used for individual RT-PCR and gene array experiments. Data are expressed as relative mRNA quantity with respect to LPS-elicited control (positive control) which is normalized to a value of 1.0 as described in Dey et al., 2006 [19] (Fig 4).

**Table 4:** Primer sequences used for real-time RT-PCR

Gene (accession number)	Forward	Reverse
IL6 (NM_031168)	5'-tagtccttcctaccccaatttcc-3'	5'-ttggtccttagccactccttc-3'
CXCL10/IP10 (NM_021274)	5'attctttaagggctggtctga 3'	5'cacctccacatagcttacagt 3'
β Actin (NM_007393)	5'-aaccgtgaaaagatgacccagat-3'	5'-cacagcctggatggctacgt-3'

IL6, Interleukin6; CXCL10/IP10, C-X-C motif ligand 10/10 kDa interferon-gamma-induced protein

### Immuno-blotting

**Figure 5:**
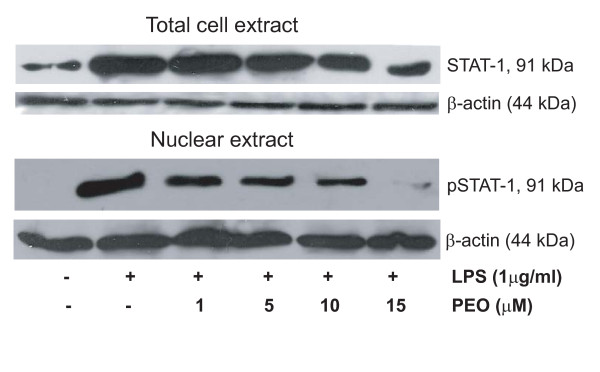
**Immunoblot analyses showing suppression of total cellular STAT1 (selected novel hit from Table 5) as well as activated nuclear pSTAT1 in response to PEO treatments in LPS-activated mouse macrophage**. Cells were pre-treated with PEO for 2 h prior to 45 min LPS-activation. Unstimulated cells and stimulated cells served as naïve and positive controls. Figure is representative of three experiments. Significance of PEO treatment with respect to positive control is discussed in results section.

For immunoblot analyses, pretreated RAW 264.7 cells were activated with LPS for 45 minutes and harvested using RIPA lysis buffer (Pierce biotechnology, Rockford, IL). For nuclear extract preparation and protein concentration determination manufacturer's (Thermo Scientific, Rockford, IL) protocols in NE-PER Nuclear Extraction Reagents and Pierce BCA Protein Assay kit were followed. Proteins (50 μg/lane) were separated by 12% SDS-PAGE and products were electrotransferred to polyvinyldene difluoride (PVDF) membranes (Amersham Biosciences Corp, Piscataway, NJ). The membranes were blocked with 5% skim milk for 1 h, washed 3 times in PBS, incubated with primary Ab (Abs against mouse βactin and STAT1 were purchased from Santa Cruz Biotechnology Inc., Santa Cruz, CA and against phosphorylated [Tyr^701^] STAT1 from Cell Signaling Technology Inc, Beverly, MA) for 1 h, washed 3 times in PBST (PBS containing 05% Tween20) and incubated with HRP-conjugated secondary Ab (Santa Cruz Biotechnology Inc., Santa Cruz, CA) for 1 h, all at room temperature. After an additional 3 washing steps, bound peroxidase activity was detected by ECL detection system (Amersham Biosciences Corp, Piscataway, NJ) (Fig 5).

### Statistical Analysis

Data are expressed as mean ± SE. Analyses for in vitro experiments were performed using Student's *t*-test. One-way ANOVA (analysis of variance) was used to determine the significance of in vivo treatments followed by post-ANOVA Tukey's Honestly Significant Difference multiple means comparison test to determine specificity of the treatments.

## Results

For convenient interpretation of the data discussed in the subsequent sections, the current paragraph highlights key features of the method section. The acute model is defined by rapid onset of severe clinical signs (sometimes interchangeably referred to as 'symptoms' in the rest of the text) that may be healed when the disease-causing agent (DSS) is withdrawn. The induction period in the acute model is shorter than in the chronic model and a higher DSS concentration is used. In the chronic model, induction of symptoms is slower but longer lasting with possible flare-up and remission cycles. DSS is administered in alternate cycles at lower concentrations for the chronic model (see Table 1 and methods). The optimal concentration of PEO (75 mg/kg) for assessing its in vivo efficacy was determined in a pilot study using a dose response administration of PEO in DSS-treated animals (data not shown). Mesalamine/5-ASA, a standard first-line therapy for mild-to-moderate UC, administered at a recommended 50 mg/kg [22], was used as a reference standard for in vivo models. In all instances, statistical significances of treatments of PEO and 5-ASA groups were compared to DSS group (Figs 1, 2, 3, Table 1). The results are presented in the order of physiological, histopathological and molecular observations. In the sections to follow, the 'water-group' and the 'DSS-group' refer to the healthy and the disease controls respectively (see Table 1 for details).

Disease activity index (DAI) (Fig 1) combined the following 5 variables (clinical signs/symptoms): change in body weight, stool consistency, occult blood in stool, rear end bleeding and inflammation (visible), and rectal protrusion. DAI was used to compare the treatment response to PEO and 5-ASA with that of the DSS group following the scale described in Table 2 [modified from [5,11]]. Rectal prolapse, one of the variables for DAI (Table 2) was observed only in the chronic colitis animals (Fig 1B) occurring with partial prolapse of the rectal lining. Therefore, the DAI shown for the acute model (Fig 1A) does not reflect this variable. Also, UC is an intermittent disease with periods of exacerbated symptoms and periods that are relatively symptoms free [11,23]. Due to the longer experimental duration of the chronic model (Table 1), the relapse-remission pattern among members of individual groups was observed (Fig 1B). In the acute model, both PEO and 5-ASA attenuated the general signs of UC after 5, 10 and 15 days of respective treatments (Fig 1A) with PEO performing notably better (PEO *p *< 0.01, 5-ASA *p *< 0.05) and comparing closely with the water (healthy) group (Fig 1A). In the chronic model, alleviation of clinical signs was not observed until after 10 days of treatment (Fig 1B). The PEO-treated group showed significant amelioration (*p *< 0.01) of symptoms on day 12 and continued to experience further relief until the end of the experiment while the 5-ASA group did not show any significant remission (Fig 1B). In addition, the PEO- treated group experienced an attenuated spike in symptoms starting from the second day of treatment (*p *< 0.01), suggesting that PEO may also prevent the relapsing nature of chronic colitis (Fig 1B).

Colon characteristics were analyzed next. Colon lengths were measured prior to flushing. The healthy water group typically had longer, non-thickened colons. In the diseased animals, the colons were often shortened (Fig 2A), likely due to loss in crypt structure and thickened due to edema formation, similar to previously reported studies with this model [24]. The differences between the healthy and the DSS-receiving groups in terms of colon lengths were less pronounced in the acute study (data not shown) as compared with the chronic study (Fig 2A), likely due to shorter induction period (5 days vs 36 days). Therefore, relief from colon shortening-effects was not observed in the acute model. In the chronic study, all of the DSS-treated groups (Table 1) had markedly shorter average colon lengths as compared with the healthy water-treated group (Fig 2A). The PEO-treated group exhibited pronounced attenuation of colon shortening (*p *< 0.05, Fig 2A) as was observed terminally.

Histology of the bowel sections of the experimental animals revealed a number of cellular changes in the colons as described in Table 3[11]. However, the population size available for histopathology study (Fig 2B) was smaller (n = 5) as compared to the observations for gross colon length (n = 10) (Fig 2A). This is because half of the 10 colons were used for biopsy samples (discussed later, Fig 3) and half were available for histopathological evaluations. The PEO-treated group had the lowest average pathological score (*p *= 0.07), closely followed by the 5-ASA group (*p *= 0.08) (Fig 2B). Qualitative differences were widespread among the bowels of both the PEO (Fig 2E) and 5-ASA (Fig 2F) groups relative to the DSS group (Fig 2D). In the bowels of the DSS treated animals, moderate to extensive infiltration of submucosa ('SM', Fig 2) and superficial muscularis ('ME', Fig 2) by a mixed population of inflammatory cells (shown in white triangles with red border, Fig 2), such as lymphocytes, plasma cells, and macrophages (all mononuclear type), was observed. In some samples the infiltration extended transmurally through the muscularis and into the serosa (data not shown). The epithelium in small patches had frequently lost the majority of their goblet cells (shown in yellow triangles with black borders, Fig 2) or ulcerated away from mucosa with intense infiltration of polymorphonuclear neutrophylic leukocytes (PMNs) beneath ulcerated areas, in contrast to the representative healthy colon section (Fig 2C). Rectal parts of the colons were generally characterized with the worst pathology including extensive infiltration, the presence of hyperplastic squamous epithelium and an increased mitotic index (data not shown). In the bowels of PEO- and 5-ASA-treated groups (Figs 2E, F) some signs of ulceration, partially inflamed submucosa, and low inflammatory cell infiltration were also observed, but to a much lesser extent and infrequently, in contrast to the DSS group (Fig 2D). Additionally, crypt structures with intact goblet cells were frequently visible (Figs 2E, F).

In order to address the biochemical basis of histological alterations, the cytokine response in the gut was evaluated by measuring the release of the pro-inflammatory cytokine, Interleukin 1β (IL1β) from colon tissue. A significant reduction in the average production of IL1β in the proximal (*p *< 0.01) and distal (*p *< 0.05) colon biopsies was observed among PEO-treated animals as compared with the DSS group. There was also a marked reduction in cytokine production in the mid colon for the PEO group (*p *= 0.06) (Fig 3). The average IL1β production was reduced by 5-ASA in the middle and distal colons.

In the present study, histopathological analyses showed infiltration by various inflammatory cells including macrophages in the diseased samples and a reduction of such infiltration in the PEO treated group. Subsequently, we performed a gene array analysis (Table 5) using the same in vitro macrophage based system to elucidate additional immune signaling genes that could be potential targets of PEO activity. Twenty one out of 84 genes (25%) induced by LPS, were suppressed three-fold or more by 10 μM PEO as compared to LPS activation alone (Table 5). LPS is a known agonist of toll-like receptor 4 signaling that plays a critical role in colitis [25]. Two genes that were down-regulated by LPS and up-regulated by PEITC were Htr2b (5-hydroxytryptamine, NM_008311) and Zap70 (zeta chain associated protein, NM_009539). LPS activation (or suppression) was separately compared to non activated levels of gene expressions (data not shown). The 21 down regulated genes represent key transcription factors and inflammatory mediators, at least 7 of which including IL6 were previously unknown to be affected by PEITC/PEO treatment (Table 5). There is one very recent report [26], however, showing in vitro down regulation of some oncogenes by PEITC in response to IL6 elicitation but effects of PEITC on IL6 expression was not reported. Out of these 7 responding genes, concentration-dependent changes in IL6, CXCL10 and STAT1 (signal transducer and activator of transcription1) expressions have been confirmed using real time RT-PCR (Fig 4) and Western Blot analyses respectively (Fig 5). These three genes were selected for further examination since their activation has been reported for human ulcerative colitis [27-29]. At 10-15 μM, a decrease in mRNA and protein levels was observed for all these three genes (*p *< 0.01) (Figs 4, 5).

**Table 5:** Genes down-regulated (≥ 3-fold vs LPS activation alone) by 10 μM PEO in macrophages

Gene name abbreviation	Gene name full	Partial Gene Ontology term http://www.geneontology.org	Fold change in response to LPS
CCL2*	Chemokine (C-C motif) ligand 2	Inflammatory response; Chemokine activity	-35.67
CSF2	Colony stimulating factor 2 (granulocyte- macrophage)	Immune response; Cytokine and chemokine mediated signaling pathway	-8.21
CSF3	Colony stimulating factor 3 (granulocyte)	Immune response; Cytokine activity	-53.69
CXCL10*	Chemokine (C-X-C motif) ligand 10	Inflammatory response; Chemokine activity	-30.05
FOS	FBJ osteosarcoma oncogene	DNA binding; Regulation of transcription	-10.90
GJA1	Gap junction membrane channel protein alpha 1	Cell-cell signaling	-19.25
IL10	Interleukin 10	Immune response; Cytokine activity	-4.95
IL1a	Interleukin 1 alpha	Inflammatory response; Cytokine activity	-291.36
IL1b	Interleukin 1 beta	Inflammatory response; Cytokine activity; Neutrophil chemotaxis; Positive regulation of IL-6 and chemokine biosynthesis	-32.83
IL6*	Interleukin 6	Signal transducer and cytokine activity	-14.39
MAPK3	Mitogen activated protein kinase 3	ATP binding; Transferase activity; Protein amino acid phosphorylation; Signal transduction; Inflammatory response; Regulation of cytokine biosynthesis; MAP kinase activity	-3.11
NFkB1	Nuclear factor of kappa light chain gene enhancer in B-cells 1, p105	DNA binding; Regulation of transcription	-21.51
NFkB2	Nuclear factor of kappa light polypeptide gene enhancer in B-cells 2, p49/p100	DNA binding; Regulation of transcription	-5.72
NFkBia	Nuclear factor of kappa light chain gene enhancer in B-cells inhibitor, alpha	Nucleus; Protein binding; Cytoplasm: Regulation of cell proliferation; Protein-nucleus import, translocation	-8.21
REL	Reticuloendotheliosis oncogene	DNA binding; Regulation of transcription	-5.76
RELb	Avian reticuloendotheliosis viral (v-rel) oncogene related B	Transcription factor activity; Intracellular; T-helper 1 type immune response	-3.78
RIPK2	Receptor (TNFRSF)-interacting serine-threonine kinase 2	Regulation of apoptosis	-5.72
SLC20a1*	Solute carrier family 20, member 1	Receptor activity; Phosphate transport	-6.81
STAT1*	Signal transducer and activator of transcription 1	DNA binding; Regulation of transcription	-4.81
TNFaip3*	Tumor necrosis factor, alpha-induced protein 3	Apoptosis; Zinc ion binding	-134.05
CD40*	CD40 antigen	Signal transduction; Immune response; Apoptosis	-46.74

* Novel hits (genes) suppressed by PEITC/PEO (previously not reported)

IL-6 signaling is mediated by STAT3 and various inflammatory diseases including UC are associated with STAT3 activation [26]. But since activity of PEITC on STAT3 activation is known [26], our subsequent investigation focused on effects of PEO on STAT1 activation and expression. Concentration dependent suppression of cytoplasmic STAT1 protein (Fig 5) and mRNA (data not shown) levels were observed. Additionally, CXCL10, a STAT1 responsive gene [30] was also suppressed by PEO in activated macrophages (Fig 4). We previously reported suppression of another STAT1 responsive gene, iNOS by PEO [19]. Therefore, we wanted to study the effects of PEO on activated (phosphorylated at Tyr^701^) STAT1 (pSTAT1). Since within seconds after phosphorylation STAT1 translocates into the nucleus for binding to the DNA of its target genes [31], we determined the levels of pSTAT1 in the nuclear fractions of the PEO treated cells. A concentration dependent attenuation of pSTAT1 was observed (Fig 5). Densitometric analysis revealed a 2.5-, 4-, and 5-fold (*p *< 0.05, with respect to LPS control) reduction in pSTAT levels by one, 5 and 10 μM PEO. At 15 μM PEO treatment, pSTAT was almost absent (*p *< 0.001) as was also observed in non-stimulated cells (Fig 5).

## Discussion

*Barbarea verna *seeds are a rich source of PEITC with the potential for providing natural protection from environmental and dietary toxins [13]. We have previously reported the anti-inflammatory properties of PEO [19]. PEO, an essential oil extracted from edible *B. verna*, containing >95% naturally occurring PEITC, was used for all experiments in the present study. Here, we present the effects of PEO in mouse ulcerative colitis models. The colitis was chemically induced with DSS and clinical signs comparable to those of human ulcerative colitis [32] were observed such as body weight loss, diarrhea, bloody stool, mucosal ulceration, and shortening of colon length [33]. DSS is directly toxic to the gut epithelial cells by weakening the integrity of the mucosal barrier [11]. When given at a higher concentration (3%) for a short duration (five days), DSS induced acute colitis, while long-term (36 days) and cyclic (alternated with tap water) administration of a lower concentration (2.5%) of DSS developed signs of chronic colitis [modification of [5,11]]. After disease induction, 75 mg/kg PEO was administered orally, once daily for two weeks. The PEO treatments significantly suppressed DSS-induced colitis in acute as well as chronic models, improving their body weights and stool consistency, as well as decreasing their intestinal bleeding (combined together as DAI, Fig 1). In addition, PEO reduced mucosal inflammation, depletion of goblet cells, and infiltration of inflammatory cells, thereby preserving colon length and structure (Fig 2). The observed attenuation of histopathological signs of colitis (Fig 2) was not as pronounced as the remission of the physiological symptoms (Fig 1) after 2 weeks of PEO-treatment. This is, however, consistent with the published report that complete healing of bowel ulceration may not always occur in parallel with clinical remission of symptoms [23]. Various clinical studies also suggest that mucosal restitution is usually measured after 8 weeks of treatments [23]. The effects of the reference drug 5-ASA (also known as mesalamine) administered at a dose of 50 mg/kg [22], produced comparable DAI to that of PEO in the acute model (Fig 1A) but were less pronounced in the chronic model (Fig 1B). It is interesting to note that although widely used as a first-line therapy for UC, 5-ASA is largely ineffective in more severe and chronic cases of the disorder [34]. For this reason and others, in recent years, the 5-aminosalicylic acid-containing pro-drug balsalazide has been the focus of attention. In a very recent metanalyses study Balsalazide was more effective than mesalamine in induction of remission, but balsalazide had no benefit compared with mesalamine in preventing relapse in the population selected. The number of patients with any adverse events and withdrawals because of severe adverse events was similar for mesalamine and balsalazide [35].

The pathophysiology of IBD is reflected by a distorted balance of regulatory cytokines [27]. We have previously reported the in vitro inhibition of IL1β, a pro-inflammatory cytokine, in immune cells by PEO [19]. In colitis tissue, the expression of IL1β increases in immune-challenged cells due to the activation of nuclear factor kappaB (NFκB) [34]. Also, local high production of IL-1β leads to tissue damage in IBD patients [1,36]. In the present study we observed a significant PEO-mediated reduction of IL1β protein in the gut tissue (Fig 3).

The NFκB and STATs are two important families of transcription factors that are activated in response to a variety of stimuli to regulate multiple cellular processes including immune response. The NFκB and STATs have distinct as well as synergistic effects on gene induction [37]. STAT proteins (7 reported in mammals) are dormant cytoplasmic transcription factors that become activated after phosphorylation by Janus kinases or other kinases in response to various stimuli including cytokines, growth factors and LPS [37]. The activated protein migrates into the nucleus and binds to specific promoter elements to regulate gene expression [31]. STAT regulated genes include inducible nitric oxide synthase (iNOS) [38], and CXCL10 [30] among others. The role of NFκB in IBD [34] as well as effect of PEITC on NFκB activity [16-18,20] are well studied. Excepting a very recent report on STAT3 [26], the effect of PEITC on STATs however has not been investigated.

An increase in STAT1 expression and activation in human UC was observed by Schreiber et al., [27]. In addition, a number of studies have also reported an increased expression of CXCL10 in the serum and mucosa of patients with UC [29,39,40]. The CXCL10 is a STAT1 responsive, CXC chemokine that promotes the migration of activated T cells. It is secreted by a variety of cell types, including macrophages [29]. The gene array analysis in the present study detected greater than three-fold suppression of STAT1 and CXCL10 mRNA by 10 μM PEO in LPS activated mouse macrophages (Table 5). Since it was previously unknown that PEITC/PEO can attenuate the expression of these genes (CXCL10 and STAT1), we further confirmed a concentration-dependent suppression of expression of these two genes by PEO (Fig 4, 5).

In macrophages we also observed a novel dose-dependent decrease of activated STAT1 (pSTAT1) in the nucleus by PEO/PEITC (Fig 5). This is an interesting observation given that macrophages are cells of monocytic lineage, and phosphorylated (p) STAT1 was mainly detected in monocytic cells and neutrophils in the inflamed mucosa of ulcerative colitis patients [27]. The inhibition of pSTAT1 by PEO is also consistent with our current and previous findings that CXCL10 and iNOS [19] mRNA are down regulated by PEO in activated macrophages. Since STAT1 regulates iNOS [38] and CXCL10 [30,41], suppression of pSTAT1 by PEO likely contributes to the downstream inhibition of iNOS and CXCL10. Based on our current observations we hypothesize that the decrease in nuclear pSTAT1 is primarily due to lower levels cytoplasmic STAT1 protein (Fig 5) that serves as the kinase substrate before translocation. We have initiated a detailed investigation into the molecular mechanism of suppression of STAT1 activation by PEO that will be described elsewhere in the future.

Published reports suggest the important role of IL-6 signaling in the development of IBD [28]. IL6 signaling is mediated by STAT3 that is also associated with colitis disease development [28]. PEITC has been shown to suppress STAT3 activation [26] but its direct effect on IL6 expression has not been reported. The gene array data in the current study show down-regulation of this proinflammatory cytokine by PEO in elicited macrophages (Table 5). We confirmed this observation using a concentration-dependent mRNA inhibition of IL6 by PEO (Fig 4).

In summary, orally administered PEO is pharmacologically active at remitting acute and chronic bowel inflammation in experimental colitis in a mammalian model system. The biological activity of PEO may be partly mediated by the inhibition of pro-inflammatory cytokines and chemokines produced by infiltrating immune cells in the inflamed gut such as macrophages, thereby attenuating signs of tissue damage. The gene expression data further indicate that PEO affects an intricate network of cellular targets of IBD including but not limited to STAT1 mediated signaling. Taken together, the physiological, histopathological and molecular findings of this study suggest that PEO might provide promising new therapeutic lead for the treatment of IBD.

## Conclusions

Orally administered PEO is pharmacologically active at remitting acute and chronic bowel inflammation in experimental colitis in a mammalian system. The physiological, histopathological and molecular findings of this study suggest that PEO might provide promising new therapeutic lead for the treatment of IBD.

## Authors' contributions

MD conceived of the study, designed all experiments, carried out major part of all experiments, interpreted all data and drafted the entire manuscript. PK helped coordinate and substantially carried out the animal experiments. DR purified the chemical compound that is being tested for biological activity and analyzed the purity of the extracted compound using GC-MS. VGP participated in animal experiment and carried out western blotting. KR analyzed the histo-pathological data and provided guidance with microscopy and imaging. DR and KR also commented on the manuscript format, grammar and content. IR provided facilities within his laboratory for some parts of the experiments that were carried out at Rutgers University. IR also commented/edited on the manuscript for intellectual property content. **All authors read and approved the final manuscript**.

## Authors' informations

**All authors have Ph.D in Biology or related fields as their highest obtained degree**.

MD was formerly Assistant Research Professor in the Biotechnology Center at Rutgers, The State University of NJ (NJ) and currently Associate Professor, Nutrigenomics Program, South Dakota State University (SD). MD specializes in Molecular Biology and Nutrient-gene interaction.

PK is a Senior Scientist at Phytomedics Inc. NJ. PK specializes in Animal Science and rodent disease models.

DR is a Research Associate in the Biotechnology Center at Rutgers University in NJ and a Principal Investigator. DR specializes in Natural Product Chemistry.

VGP was formerly a postdoctoral Associate in Rutgers University and is currently working as a postdoctoral researcher at University of Cincinnati, OH.

KR is a Professor in the Department of Pharmacology and Toxicology at Rutgers University, NJ.

IR is a Professor in the Department of Plant Biology and Pathology at Rutgers University, NJ.
